# Case report: A rare case of oral sebaceous carcinoma in the upper lip

**DOI:** 10.3389/pore.2024.1611968

**Published:** 2024-11-08

**Authors:** Yousef Katib, Murad Essatari

**Affiliations:** ^1^ College of Medicine, Taibah University, Madinah, Saudi Arabia; ^2^ King Abdulaziz Medical City, Prince Noorah Oncology Center, Jeddah, Saudi Arabia

**Keywords:** sebaceous carcinoma, oral cavity, lip, immunohistochemistry, treatment, oral cavity

## Abstract

Sebaceous carcinoma (SC) is a rare aggressive malignant tumor that originates in the adnexal epithelium of the sebaceous gland. While occurrences on the lips are extremely uncommon, there have been a few reported cases in the literature. Our case involves a 47-year-old smoker male who presented with a painless, non-mobile lesion on his upper lip that had been present for 12 months. Upon clinical examination, an ulcerated, exophytic, and irregularly shaped mass was observed on the upper lip. No other intraoral lesions were found. An incisional biopsy was performed, revealing a malignant tumor with a nodular pattern consisting of basaloid cells with obvious sebaceous differentiations and frequent mitoses. The neoplastic cells tested positive for broad-spectrum cytokeratin (AE1-AE3), epithelial membrane antigen (EMA), and P53, while testing negative for S-100 and carcinoembryonic antigen (CEA). Based on these results, a diagnosis of SC of the upper lip was made. This case report and review aimed to describe the histogenesis, unique clinicopathological features, and current treatment options for SC.

## Introduction

Sebaceous carcinoma (SC) is a rare malignant neoplasm that arises from the sebaceous glands and is associated with sebocytic differentiation [1]. Approximately 400 cases have been documented in the literature to date. Given its rarity and lack of widely recognized histopathological classification, the presentation of SC poses a diagnostic challenge [2]. This tumor typically affects the head and neck region, particularly the eyelid, but can occur in any part of the body [2–4]. It is often misdiagnosed as basal cell carcinoma (BCC) or squamous cell carcinoma (SCC), which are more prevalent forms of skin cancer [5]. SC can also resemble other sebaceous tumors such as adenomas, sebaceomas, and hyperplasia [6]. While there have been rare reports of SC originating from the parotid or salivary gland [7], intraoral SCs are exceptionally uncommon, despite the presence of sebaceous adenomas in the oral cavity originating from sebaceous glands [7]. Around 80% of adults have a sebaceous gland in the oral cavity mucosa known as “Fordyce granule,” considered a normal anatomical component [8]. These granules are described as small, whitish-yellow papules on the oral mucosa. However, in some instances, these granules may undergo malignant transformation, leading to SC [9]. The first reported case of intraoral (IO) SC was in 1991 [10].

The upper eyelid is the most common site for SC. There are approximately 16 reported cases of intraoral SC, six of which originated from the lip as indicated in (Table 1) [2, 4, 10, 13, 15]. While the exact cause of SC remains unknown, various factors have been linked to an increased risk of its development. Inherited genetic mutations, such as those seen in Muir–Torre Syndrome, a variant of Lynch syndrome, have been associated with SC. These mutations impact DNA mismatch repair genes (*MSH2, MSH6, MLH1*), resulting in tumors characterized by microsatellite instability [16]. Due to the lack of standardized imaging protocols for staging SC, histopathological examination of excised or biopsied lesions is currently the most reliable diagnostic method [17]. SC is considered an aggressive tumor with the potential for local extension and distant metastasis [18, 19], highlighting the importance of early detection for disease management. Accurate staging, using TNM staging system, is critical in the management of SC because it helps determine the extent of cancer spread, guides treatment decisions, and provides prognostic information.

**TABLE 1:** Reported cases of sebaceous carcinoma (SC) of the lip in the literature since 2005.

	Authors	Year	Country	Sex	Age	Smoker	Site	Size	Treatment	FU
1	Alawi [3]	2005	United States	M	66y	Yes	Upper lip (Intra-oral “IO”, Mucosal)	1.5 cm	Excision	1y
2	Innocenzi [11]	2005	Italy	F	68y	No	Upper lip (IO, Mucosal)	2.0 cm	Excision	3y
3	Greenall [12]	2015	United Kingdom	M	81y	N/A	Upper lip (Extraoral “EO”, skin)	nil	Palliative + RT	Nil
4	Aray [13]	2015	Japan	M	61y	Yes	Lower lip (EO, skin)	0.15 cm	Excision	2y
5	Di Cosola [14]	2021	Italy	M	71y	Yes	Lower lip (EO, skin)	1.8 cm	Excision	3y
6	Benedict [15]	2022	United States	F	7y	No	Upper lip (IO, mucosal)	1.2 cm	Excision	1.5y
7	Present case	2023	KSA	M	47y	No	Upper lip (EO, Skin)	1.5 cm	Excision + RT	1y

The optimal treatment approach of SC involves local surgical excision and margins clearing. Neck dissection with examination of regional or sentinel lymph nodes should be considered, particularly in cases of SC affecting the eyelid [2, 18, 19]. Resection with 5 mm surgical margins is recommended to prevent local recurrence [20]. The use of post-surgical radiotherapy (RT) or chemotherapy (ST) is controversial, especially for non-eyelid cases. RT may be utilized as an alternative for patients who decline surgical intervention [18, 21]. Some studies reported a notable recurrence rate associated with RT [22]. For this reason, RT is typically considered as an adjuvant treatment for advanced cases [22]. The prognosis of SC is influenced by various factors, including its location, size, clinical stage, and the type of treatment received [19].

We herein report a case of SC of the upper lip in a 47-year-old smoker. Our case is considered as the 7th reported case in the literature.

## Case description

A 47-year-old male soldier presented with a painless, nonmobile ulcerated lesion in the left angle of his upper lip that had been present for 1 year. Despite being a heavy smoker, he had no significant past medical history or history of facial trauma. There was no family history of cancer. Upon clinical examination, a non-tender, non-mobile soft nodule was observed in the left angle of his upper lip. The mass appeared lobulated, red in color, with an irregular border and occasional discharge. No other intraoral masses were detected. Facial nerve integrity was confirmed during the examination. A flexible nasolaryngoscopy was performed, revealing bilaterally mobile vocal cords with no abnormalities. Initial potential diagnoses included basal cell carcinoma (BCC), squamous cell carcinoma (SCC), and a salivary gland tumor.

An incisional biopsy of 1 cm in size was performed through the mass. After proper fixation in formalin, the specimen was submitted entirely for processing and embedding in Paraffin. Multiple micron-thick serial sections were obtained from formalin-fixed, paraffin-embedded surgical specimens. Multiple serial sections were stained with hematoxylin–eosin and examined.

Immunohistochemistry was performed on the remaining serial sections using the labeled streptavidin-biotin complex system (Ventana) to study the expression of broad-spectrum cytokeratin, epithelial membrane antigen (EMA), S-100 protein, carcinoembryonic antigen (CEA), P53 and Ki67, using the following primary antibodies: anti-CK (clone AE1-AE3, 1:100), anti-EMA (clone E29, 1:100), anti-S100 (clone 4c4.9), anti-p53 (clone DO-7; 1:100), anti-KI67 (clone 30-9) and anti-CEA (II-7; 1:50).

**FIGURE 1:**
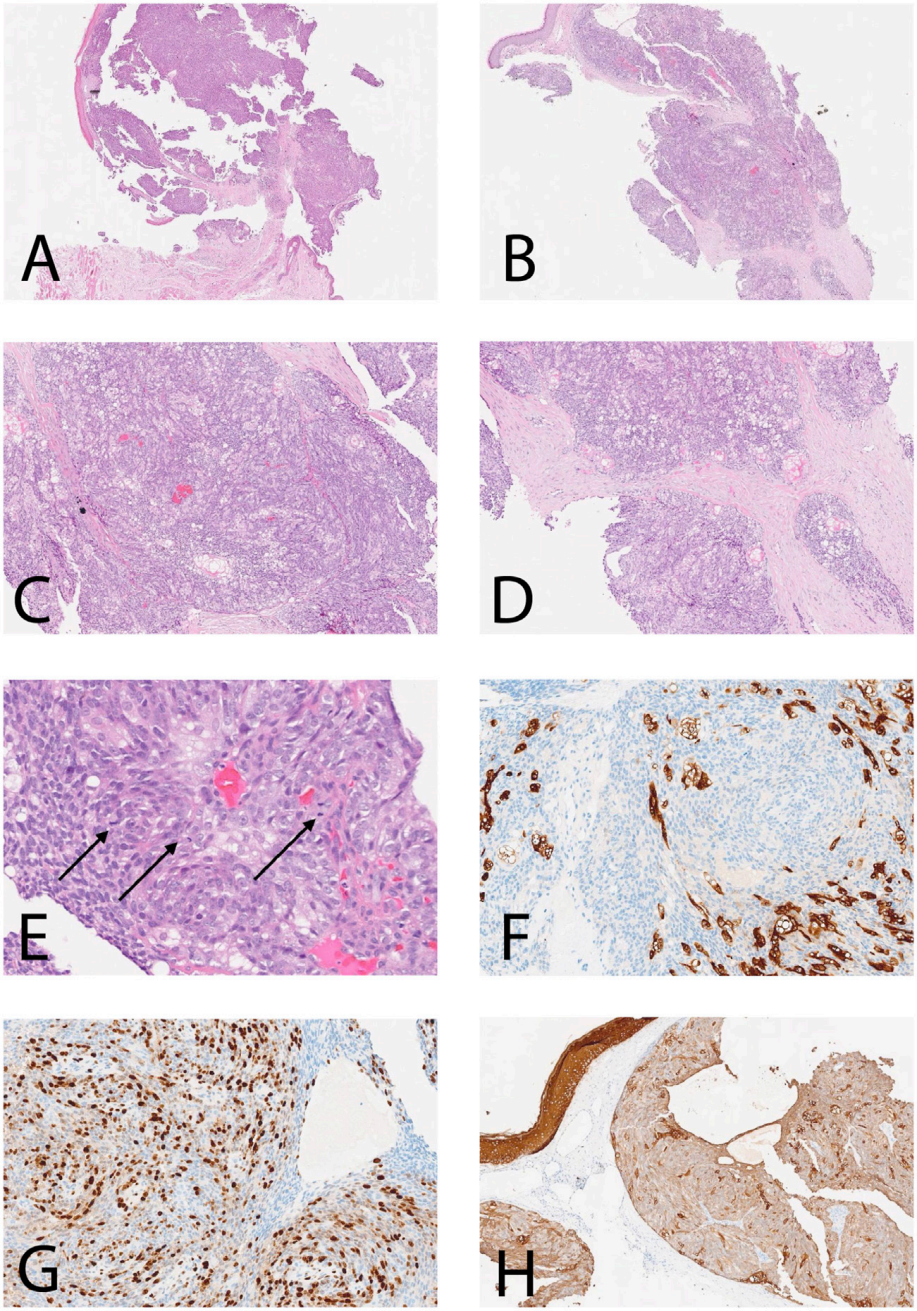
Histological features and IHC of the current case. **(A–D)** Dermal-based SC with nodular pattern [**(A, B)**: ×5; **(C, D)**: ×25]; **(E)** H&E section shows SC with brisk mitotic figures (*arrows*) (H&E ×40); **(F)**The neoplastic cells are positive for CK AE1-AE3 (×40); **(G)** High proliferative index Ki-67 estimate to be more than 60% in focal areas (×40); **(H)** Neoplastic cells are partially positive for EMA (×25).

The H&E sections show dermal-based tumor arranged in a nodular pattern and composed of basaloid looking cells with sebaceous differentiation (multivacuolated cells), with moderate polymorphism, atypia, and frequent mitoses (Figure 1A). No peripheral palisading or squamous differentiation seen. By immunohistochemistry, the neoplastic cells revealed positivity for broad-spectrum cytokeratin (AE1-AE3) (Figure 1B) and partial positivity for Epithelial membrane antigen (EMA) (Figure 1C) with increased expression of protein P53 and high proliferative index expressed by KI67 estimated about 50% in hotspot areas (Figure 1D) while they were negative for S100 and carcinoembryonic antigen (CEA). Based on the morphological and immunophenotype features, the diagnosis of sebaceous carcinoma was confirmed.

Subsequently, the patient underwent a left lip-wide local excision with reconstruction using a local flap and left-modified radical neck dissection. Lip excision measured 2 cm × 1.6 cm × 1 cm was received in formalin container. After a proper fixation, tissue was serially sectioned, and showed a polyploid exophytic white lesion measuring 1.5 cm × 0.7 cm × 0.5 cm. On histological examination, H&E sections confirmed the diagnosis of sebaceous carcinoma with depth of invasion measuring 1.15 cm. The surgical margins around the tumor were free, and no lymphovascular or perineural invasion was identified. Of the fifty-nine lymph nodes removed during neck dissection, none showed signs of metastasis.

The patient underwent a clinical, radiological staging workup and gastrointestinal screening with scopes, revealing no abnormalities or distant metastasis. The case was reviewed at the multidisciplinary tumor board, and due to the high risk of tumor recurrence, adjuvant RT was recommended. The patient completed a course of adjuvant RT totaling 60 GY over 30 sessions. Three months post-RT, magnetic resonance imaging (MRI) scan indicated no tumor recurrence.

## Discussion

Sebaceous features can be detected in the normal salivary glands, and glands exhibiting sebaceous characteristics are commonly located in the oral cavity. However, SC is uncommon in these anatomical locations [4, 14]. SC is categorized into ocular and extraocular, with the extraocular form further divided into extraoral or intraoral SC. While ocular SC originates from the meibomian glands in the tarsus, and/or the Zeiss glands of the eyelashes, the origin of extraocular SC is debated as there is no conclusive evidence of carcinoma arising from pre-existing sebaceous glands [23]. It is theorized that extraocular SC develops from intradermal cells [23]. Ocular SC is noted to be similar in aggressiveness to extraocular SC based on the literature findings [17]. A study examining 2,422 cases of SC over a 10-year period revealed that none of the orbital SC cases metastasized to the locoregional lymph nodes among all extraocular head and neck SC cases [18]. In contrast, two out of five cases of extraocular SC metastasized to nearby lymph nodes [18].

Extraocular intraoral SC typically affects the lips, gums, tongue, floor of the mouth, and other oral structures. A comprehensive literature review identified only 18 cases of extraocular intraoral SC. The reported locations included the floor of the mouth (12%), buccal mucosa (31%), palate (6%), upper labial mucosa (25%), gingiva (12.5%), and tongue (12.5%) [23]. The average age of most cases was approximately 64 years, with sizes ranging from 1.5 to 4.5 cm. In 6 out of 16 cases (37.5%), adjacent sites were involved or metastasis to lymph nodes and lungs occurred. Most reported cases had a significant history of long-term smoking. Among the 18 reported intraoral SC cases, 6 cases involved the lips, reported in the period between 2005 and 2023 (Table 1). Most of the cases were male smokers and in their 70s, with no reported instances of tumor recurrence. In our case, the diagnosis of cutaneous SC of the lip was established based on clinical observation and histological examination of the mass. The tumor exhibited ulceration, exophytic growth, irregular shape, and histological features consistent with malignant neoplastic cells displaying sebocytic differentiations. To the best of our knowledge, this case represents an exceptionally rare occurrence of SC in the literature since 2005. Although SC is often linked with multiple sebaceous neoplasms in Muir-Torre syndrome [14], our case is unique in that SC appeared exclusively on the lip.

SC should be differentiated from BCC and SCC, which are the most common malignant skin tumor types [24]. BCC is composed histologically of nests of basaloid cells with peripheral palisading [25]. It is associated with a fibromyxoid stroma and cleft formation between tumor lobules and stroma and rarely exhibits sebaceous differentiation [25–27]. SCC shows an intraepidermal component, pagetoid spread, with areas with conventional features like keratinization and keratin pearls. However, no peripheral palisading, clefting, myxo-inflammatory stroma, or sebaceous differentiation was seen [11, 12, 17, 26, 27]. In some cases, it is crucial to distinguish SC from SCC with hydropic degeneration, as the latter displays a distinct growth pattern, as mentioned above. Furthermore, there is evident atypical cell proliferation in SCC, with neoplastic cells expressing high Ki-67 along with CK and EMA positivity but lacking S-100 expression [11, 12, 17, 26, 27].

The optimal treatment for SC remains uncertain, with options including wide excision, pre- and postoperative RT, and CT. However, the choice of treatment is determined by factors such as the patient’s overall health, the location and extent of the tumor, TNM staging, and the presence or absence of metastasis. While the use of CT and RT is still a topic of debate, there have been instances where RT has shown benefits, particularly in cases where surgery is not feasible or in palliative care situations. In our particular case, the patient underwent wide local excision and lymph node dissection, followed by adjuvant therapy. A full course of adjuvant RT was completed, and the patient is currently alive, undergoing regular follow-up appointments with both surgical and oncology teams every 3–6 months. Routine imaging studies are also conducted to monitor for any signs of recurrence.

## Conclusion

A rare case SC affecting the upper lip is outlined. A male patient presented with a chronic, painless and non-metastatic poorly differentiated SC. The tumor was successfully removed through complete excision, followed by adjuvant RT with no postoperative CT. While the patient expressed relative satisfaction with the treatment received, it is worth noting that treatment options for oral SC are constrained.

## Data Availability

The original contributions presented in the study are included in the article/supplementary material, further inquiries can be directed to the corresponding author.
